# Obturation of Simulated Internal Resorption Cavities With Four Different Techniques and a Hydraulic Root Canal Sealer: A Comparative Stereomicroscopic Evaluation

**DOI:** 10.7759/cureus.76443

**Published:** 2024-12-26

**Authors:** Pavan Varanasi, Muralidasan Kalaivani, Janani Balachandran, Pooraninagalakshmi J, Mitthra Suresh, Indumathi Manoharan

**Affiliations:** 1 Conservative Dentistry and Endodontics, Sri Kamakshi Sai Dental Hospital, Tirupati, IND; 2 Conservative Dentistry and Endodontics, Sree Balaji Dental College and Hospital, Bharath Institute of Higher Education and Research, Chennai, IND; 3 Conservative Dentistry and Endodontics, Sri Ramaswamy Memorial (SRM) Kattankulathur Dental College and Hospital, Sri Ramaswamy Memorial Institute of Science and Technology (SRMIST), Chennai, IND; 4 Conservative Dentistry and Endodontics, Sri Ramachandra Dental College and Hospital, Sri Ramachandra Institute of Higher Education and Research, Chennai, IND

**Keywords:** bioceramic sealer, endodontic treatment, hydraulic condensation, image j software, internal resorption, obturation technique

## Abstract

Aim

To compare the quality of obturation using WVC (warm vertical compaction), CLC (cold lateral compaction), injectable (iFill), and Thermafil (GuttaCore) techniques, along with hydraulic condensation of bioceramic (BC) sealer, and evaluating the percentage of gutta-percha (GP), sealer, and voids in simulated internal resorptive lesions, using an advanced stereomicroscope with ImageJ software (National Institutes of Health, Bethesda, MD, USA).

Methods and material

In this study, 40 freshly extracted maxillary incisors were collected, and endodontic instrumentation was done to working length using hand K-files. Simulated internal resorption cavities were created in the middle-apical third of the roots after horizontal sectioning and were re-cemented. The root canals were obturated using four techniques: WVC, CLC, iFill, and Thermafil, along with BC sealer. After one week, the samples were resectioned, the surfaces were photographed, and the percentage of GP, sealer, and voids in resorptive cavities was calculated using an advanced stereomicroscope and ImageJ software, respectively. The data were statistically analyzed using one-way analysis of variance (ANOVA) and post-hoc Tukey tests.

Results

The iFill approach had the highest total filled area in the simulated resorptive region (96%), followed by WVC (92%), CLC (91%), and GuttaCore (77%). CLC had a notably high percentage of sealers (46%), compared to WVC (40%) and Thermacore (41%). With 23% of voids, the GuttaCore technique was considered the least successful.

Conclusions

Warm GP condensation techniques, combined with hydraulic sealer in filling artificial resorption cavities, outperformed the other obturation procedures.

## Introduction

Internal root resorption (IRR) is a condition of the vital pulp, initiated by an inflammatory process of unknown etiology, that leads to dentinoclastic activity followed by resorption in the root canal. Trauma was considered an antecedent in most cases [1]. This pathological process causes complex, irregular resorptive lacunae in the root canal, filled with organic debris and bacteria [2].

During endodontic management of IRR, complete instrumentation and disinfection of the root canal contents are challenging, as conventional root canal instruments cannot reach the root canal irregularities in IRR, which necessitate proper irrigation to dissolve and flush out the remnants of resorptive lacunae [3]. Achieving total obturation and a hermetic seal of the resorptive lesion is critical for the long-term clinical outcome of IRR, as tightly condensed gutta-percha (GP) might block dentinal tubular openings, resulting in improved sealing and the prevention of additional microbial invasion by entombing surviving microbes [2].

Previous studies have compared and concluded various obturation techniques, along with eugenol and resin-based sealers, to efficiently obturate simulated internal resorptive cavities by evaluating the quality of obturation mass and voids [1-6]. Nevertheless, the ideal method for obturating IRR without voids has not yet been ascertained. The inability of GP to adhere to dentin walls, its shrinkage after cooling, and a significant percentage of sealers in the obturation of resorptive lesions in these studies, show that applying root canal sealer is crucial for reducing voids in IRR [7].

Hydraulic calcium silicate-based sealers (HCSBS), like bioceramics (BC) and mineral trioxide aggregate (MTA), are considered ideal, ascribing to their superior flow and sealing properties [8]. Hydraulic pressure achieved during the placement of GP in a canal filled with BC sealer can drive the sealer three-dimensionally to achieve a void-free seal in complex canal anatomies [9]. Existing studies have evaluated the obturation of IRR using three-dimensional radiography, which fails to differentiate GP and sealer [10,11].

Hence, the purpose of this stereomicroscopic evaluation was to comparatively analyze the percentage of GP, sealer, and voids quantitatively, using image analysis software in simulated IRR defects obturated with four clinically recommended obturation techniques and a hydraulic BC sealer. The null hypothesis was that there was no significant difference between these obturation techniques, along with BC sealer, in terms of obtaining a void-free obturation of IRR.

## Materials and methods

In this study, 40 recently extracted human maxillary central incisors with completed root closure were used, with a G*Power software (Heinrich-Heine-Universität Düsseldorf, Düsseldorf, Germany) of 90%. The teeth were cleaned ultrasonically for superficial removal of calculus and debris, disinfected with thymol solution, and preserved in saline at room temperature.

Endodontic access cavities were made for all the teeth; the working length was determined using the visual method with a no. 10 K file (Mani Inc., Utsunomiya, Japan), which was placed inside the root canal until the file tip was visualized at the apical foramen using a magnifying glass, followed by adjustment of the stopper to the coronal reference point. A vernier caliper was used to measure the distance between the file tip and the stopper. The working length was determined by reducing 1 mm from this measurement, as the apical constriction is 0.5 to 1 mm short of the apical foramen. The canals were instrumented using the stepback technique under copious irrigation with 3.25% sodium hypochlorite solution (Prime Dental, Thane, India) and saline (Klokter Lifesciences Pvt. Ltd., Pune, India) up to a master apical file size of #50, and apical tugback was verified with the corresponding master cone (Dentsply Maillefer, Ballaigues, Switzerland). Horizontal sectioning of the roots was done 7 mm coronal to the apex with a fine diamond disc, followed by the creation of a semicircular cavity of 2 mm using a high-speed round (no. 6) bur (Mani Inc.) on each half of the tooth.

A small drop of cyanoacrylate adhesive (Pidilite, Mumbai, India) was applied gently to the adjoining dentin surface using the tip of a dental explorer. The relevant portions were then bonded together using the guiding mark, while maintaining canal patency by placing the master cone in the canal, and were confirmed using digital intra-oral periapical radiographs (IOPARs) both in the buccolingual and mesiodistal directions. Based on the obturation technique, four groups (n = 10) were divided by randomization.

Group I. Cold lateral compaction (CLC)

Premixed BC sealer (Angelus Dental, Londrina, Brazil) was placed within the root canal with a lentilospiral (Mani Inc.), and then the master cone was minimally coated with sealer and inserted into the canal. Lateral compaction was done using accessory cones coated with sealer and standardized finger spreaders. The coronal portion of GP was seared with a warm instrument up to the orifice level and well compacted.

Group II. Warm vertical compaction (WVC)

Following the hydraulic condensation of BC sealer and master cone placement, #40 and #50 stainless steel hand pluggers (Etchenem) were used for downpacking the master cone and segmental (3 mm) vertical compaction of the remaining part of the root canal, respectively, such that GP flows and fills the canal spaces and irregularities.

Group III. Injectable (iFill)

Following BC sealer and master cone placement, the master cone was sheared off at the orifice with a hot instrument. Downpacking was done up to 3 mm from the apex using a suitable plugger tip, heated to 200°C with an iFill pen (Denjoy Dental Co., Ltd., Changsha, China). Backfilling was done incrementally by injecting GP using an iFill gun set at 200°C. At the orifice level, the mass of GP was firmly compacted with a #50 plugger.

Group IV. GuttaCore

After hydraulic placement of BC sealer in the canal walls, the GuttaCore (Dentsply Maillefer) obturator was softened in a ThermaPrep oven (Dentsply Maillefer) for a minimum of 10 seconds and slowly inserted into the canal, up to 0.5 mm short of the working length. The core handle was then separated at the root canal orifice.

Following obturation using four different techniques, access cavities were restored with Glass Ionomer Cement (GC Corporation, Bunkyo, Japan), and the samples were stored in artificial saliva (ICPA Laboratories, Mumbai, India) for seven days at normal room temperature.

**Figure 1:**
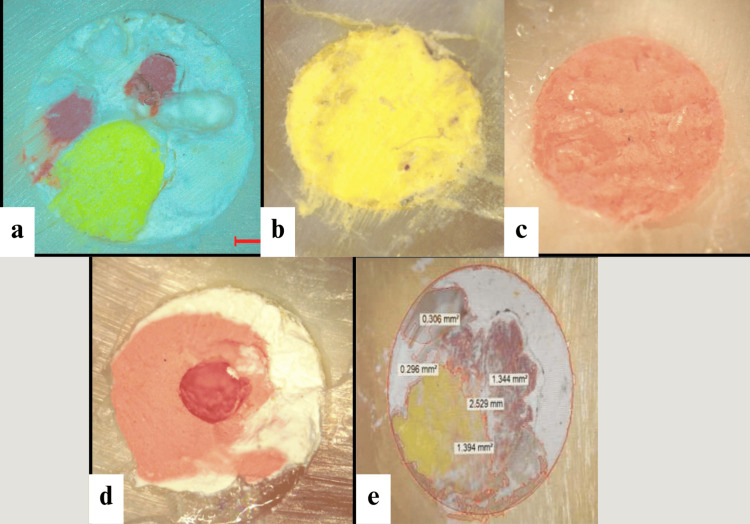
Stereomicroscopic images of study groups (a) CLC Technique, (b) WVC technique, (c) Injectable GP Technique, (d) Guttacore technique, (e) Quantitative analysis of stereomicroscopic images using Image J software CLC, Cold lateral compaction; WVC, Warm vertical compaction; GP, Gutta-percha

The IOPAR of each tooth was taken both in the buccolingual and mesiodistal directions, re-sectioned at the same level as the previous sectioning, and examined under the high-end stereomicroscope (Leica Microsystems, Rotkreuz, Switzerland). Photographic images were recorded (Figure 1).

The photographs were added to computer-based image analysis software, ImageJ (National Institutes of Health, Bethesda, MD, USA), which calibrated and calculated the amount of GP, sealer, and voids in percentages (Figure 1) within the simulated IRR cavities.

IBM SPSS Statistics for Windows, Version 20 (Released 2011; IBM Corp., Armonk, NY, USA), was used to statistically analyze the results attained from the present study.

## Results

The difference in mean percentage and standard deviation ratios of GP and sealer, GP, sealer, and voids for various study groups can be observed in Table 1. Among the study groups, injectable GP showed the highest percentage of GP + sealer (91.14 ± 1.51), GP (63.33 ± 1.19), and the lowest percentage of voids (4.03 ± 0.89) and sealer (32.66 ± 1.50). CLC had the highest sealer percentage (46.10 ± 3.01), followed by Thermacore (40.84 ± 3.57) and WVC (39.43 ± 2.05), with a statistically insignificant difference between the two groups. On the contrary, the GuttaCore technique showed a significantly higher percentage of voids (23.33 ± 2.41) and a lower percentage of GP + sealer (76.64 ± 2.44).

**Table 1:** Descriptive analysis comparing the percentage of area covered by GP, sealer and voids in four obturation groups CLC, Cold lateral compaction; WVC, Warm vertical compaction; GP, Gutta-percha; SD, Standard deviation; SE, Standard error

Materials	Groups	Minimum (%)	Maximum (%)	Mean	SD	SE
GP + sealer	CLC	88.48	93.33	91.14	1.51	0.48
	WVC	90.16	94.66	92.34	1.39	0.44
	Injectable	94.30	97.30	95.96	0.89	0.28
	GuttaCore	72.78	80.10	76.64	2.44	0.77
GP	CLC	41.78	48.38	44.76	1.94	0.61
	WVC	50.60	55.07	52.91	1.37	0.43
	Injectable	61.00	65.26	63.33	1.19	0.38
	GuttaCore	31.21	38.16	35.76	2.09	0.66
Sealer	CLC	41.10	51.55	46.10	3.01	0.95
	WVC	37.02	42.45	39.43	2.05	0.65
	Injectable	30.02	34.98	32.66	1.50	0.48
	GuttaCore	34.62	45.78	40.84	3.57	1.13
Void	CLC	6.67	11.52	8.86	1.51	0.48
	WVC	5.34	9.84	7.66	1.39	0.44
	Injectable	2.70	5.70	4.03	0.89	0.28
	Guttacore	19.90	27.22	23.33	2.41	0.76

One-way analysis of variance (ANOVA) revealed a significant disparity among the groups, statistically, with a p-value of 0.001. Based on the determined F-value, the null hypothesis was rejected, indicating that the variation observed between the groups is not attributable to random variability (Table 2).

**Table 2:** Comparison of the mean percentage area of GP + sealer using one way ANOVA *p < 0.0001 (statistically significant) ANOVA, Analysis of variance; GP, Gutta-percha

	Sources of variation	Sum of squares	df	Mean square	F-value	p-value
GP + sealer	Between groups	2168.72	3	722.91	263.7050	0.0001*
	Within groups	98.69	36	2.74		
	Total	2267.41	39	-		
GP	Between groups	4136.37	3	1378.79	482.9360	0.0001*
	Within groups	102.78	36	2.86		
	Total	4239.15	39	-		
Sealer	Between groups	919.04	3	306.35	43.3370	0.0001*
	Within groups	254.48	36	7.07		
	Total	1173.52	39	-		
Voids	Between groups	2161.99	3	720.66	266.5620	0.0001*
	Within groups	97.33	36	2.70		
	Total	2259.32	39	-		

The mean difference values in pairwise analysis of the study groups, using the post-hoc Tukey test (Table 3), demonstrated a statistical difference between different obturation techniques (p < 0.005) in total filled area, GP, sealer, and voids.

**Table 3:** Pairwise comparison of study groups by post-hoc multiple Tukey’s test *p < 0.0001 (statistically significant); I-J indicate mean difference between groups CLC, Cold lateral compaction; WVC, Warm vertical compaction

Groups		Total filled area (GP + sealer)		GP		Sealer		Voids	
(I)	(J)	(I-J)	p-value	(I-J)	p-value	(I-J)	p-value	(I-J)	p-value
CLC	WVC	-1.20	0.3830	-8.14	0.0001*	6.67	0.0001*	1.20	0.3770
	Injectable	-4.82	0.0001*	-18.57	0.0001*	13.44	0.0001*	4.83	0.0001*
	GuttaCore	14.50	0.0001*	9.00	0.0001*	5.26	0.0001*	-14.47	0.0001*
WVC	Injectable	-3.63	0.0001*	-10.42	0.0001*	6.77	0.0001*	3.63	0.0001*
	GuttaCore	15.69	0.0001*	17.15	0.0001*	-1.41	0.6420	-15.66	0.0001*
Injectable	GuttaCore	19.32	0.0001*	27.57	0.0001*	-8.18	0.0001*	-19.29	0.0001*

## Discussion

Internal resorption is most commonly prevalent in the middle third of maxillary incisors [3]. Obturation of such lesions at the middle-apical junction is a challenge for the clinician due to inaccessibility, pressure differences while compacting, and ease of filling in the near zone [3]. Hence, this clinical scenario was simulated in this study.

The existence of voids encourages microleakage and creates a habitat for bacteria to flourish, which promotes re-infection. Open voids through dentinal tubules can connect the periodontal ligament space to the root canal, lowering the quality of the eventual obturation [12]. Radiographic techniques employed in earlier research to evaluate the obturation of resorptive regions were insufficient in assessing the quality of obturation, since it is challenging to measure voids three-dimensionally and nearly impossible to distinguish between GP and sealer [13].

In this study, the proportion of GP, sealer, or voids in the simulated resorptive cavity was calibrated quantitatively using an image analysis application, ImageJ. It is a public domain software for volumetric analysis that is accurate and cost-effective for computing, measuring, and comparing the area and pixel value of selections defined by the user. It can generate density histograms and profile plots with the data it analyzes, without the need for expensive devices [14].

Several studies have reported the quality of various obturation techniques using eugenol and resin-based sealers, which showed a greater percentage of voids [2-5]. The major drawbacks of these traditional sealers are shrinkage after setting and solubility when exposed to tissue fluids. These dimensional changes can potentially lead to voids and microleakage in root canals [15,16]. To counter these limitations, a higher proportion of core materials (GP) is traditionally chosen, with the sealer proportion kept as low as feasible in root canal filling [15,17]. However, the hydraulic condensation of BC sealer in the current study is considered superior to other sealers in sealing root canals, due to its hydrophilic properties that aid in better flow and slight expansion. This is crucial in achieving a total filling without voids and a hermetic seal by preventing micro-infiltration in the root canal. Compared to other conventional sealers, HCSBS can form a mineralization reaction that improves the fracture resistance of the root by enhancing the hardness of the dentin and its elastic modulus [8,17-21]. They also exhibit improved cell viability and better fibroblast adherence, thus interacting with periapical tissue stem cells to induce biological sealing and initiate the healing process [22,23]. 

Out of all the methods employed in this investigation, injectable GP (iFill) demonstrated uniform obturation of GP with fewer sealants and voids, thereby rejecting the null hypothesis in the current study. Numerous studies in the literature have proposed the use of a thermoplasticized approach, in which beta-phase GP is incrementally injected in small aliquots and condensed into the root canal [1-7,22]. This thermoplastic injectable GP allows superior flow of core material and adaptation into complex canal anatomies, and the phase transformation results in a homogeneous core in obturation [24,25].

The second technique with the lowest percentage of voids was WVC. Its high sealing ability is attributable to its hydrodynamic nature, which involves down-packing and condensing the master cone, along with increments of GP, resulting in a more homogeneous filling with good adaptability to root canal walls [1-4,6,26].

Due to insufficient pressure, poor adaptation, or a mismatch between the tapers of the spreader, GP, and canal, CLC was less successful in obturating resorptive cavities. This caused gaps between the GP cones, which were most likely filled with sealers [1-4,27].

The Thermafil (GuttaCore) technique had the greatest void percentage of all the techniques. These findings were comparable to those of Gencoglu et al. [2]. More voids could be the result of the warmed GP material encircling the core, failing to fill the space in the resorptive area. The net force is not sufficient, as it is non-condensable by pluggers and stainless-steel lateral condensers due to the polymer core, which does not allow it to do so. The quantity and degree of fluidity of the GP material around the core are variable, which may alter the quality of obturation [2,4].

Although the study assessed the quality of several obturation techniques in IRR, the evaluation was limited to a specific region of focus (middle-apical third sections), rather than encompassing the complete canal obturation. The in vitro nature of our study, the minimal hard tissue loss resulting from the tooth sectioning to create voids, and the use of cyanoacrylate glue for its reattachment account for the limitations of our research.

## Conclusions

The study concludes that void-free root canal filling was not achieved by any of the obturation techniques. Nevertheless, the simulated internal resorptive cavities were successfully sealed with the least amount of voids when injectable GP was used, and it is considered the most recommended obturation technique. Other GP condensation techniques, such as WVC and CLC, showed satisfactory sealing of IRR lesions, with a noticeably higher percentage of sealer in CLC. However, the clinical application of these techniques, in conjunction with hydraulic condensation of BC sealer, is acceptable, considering its superior sealing properties in root canals. The GuttaCore technique is least recommended for these clinical scenarios.
